# Endogenous opioid inhibition of proliferation of T and B cell subpopulations in response to immunization for experimental autoimmune encephalomyelitis

**DOI:** 10.1186/s12865-015-0093-0

**Published:** 2015-04-24

**Authors:** Patricia J McLaughlin, Daniel P McHugh, Marcus J Magister, Ian S Zagon

**Affiliations:** Department of Neural & Behavioral Sciences, Pennsylvania State University College of Medicine, 500 University Drive, MC H109 Hershey, PA USA

**Keywords:** Low dose naltrexone, OGF, T cell proliferation, Experimental autoimmune encephalomyelitis, Multiple sclerosis

## Abstract

**Background:**

Experimental autoimmune encephalomyelitis (EAE), an animal model of multiple sclerosis, is induced by immunization of mice with myelin oligodendrocytic glycoprotein (MOG_35-55_) injections, and after 9 days, mice develop behavioral signs of chronic progressive EAE. Proliferation of T and B cells located in peripheral lymph tissues such as spleen and inguinal lymph nodes of C57BL/6J mice are stimulated. The opioid growth factor-opioid growth factor receptor (OGF-OGFr) axis has been shown to effectively limit progression of chronic EAE when mice are treated at the time of induction or at time of established disease. In addition to repressed behavioral profiles, spinal cord neuropathology is diminished in mice treated with OGF or low dosages of naltrexone (LDN). However, there is little or no information on peripheral lymphocyte dynamics following immunization of mice with MOG antigen and treatment with OGF or LDN.

**Methods:**

Six-week old female mice were immunized with MOG35-55 and were injected intraperitoneally with OGF or a low dosage of naltrexone (LDN) beginning at the time of immunization; saline-injected immunized mice served as controls. Normal mice received saline for all injections. Periodically over a 2 week period, spleens and inguinal lymph nodes were removed, total lymphocytes counted, and subpopulations of CD4+ and CD8+ specific T-cells, as well as B lymphocytes, were determined by flow cytometry. On day 15 of treatment, lumbar spinal cord tissue was removed; CNS lymphocytes isolated, and assayed for Th1, Th2, and Th17 markers by flow cytometry.

**Results:**

Exogenous OGF or endogenous OGF following LDN suppressed T and B lymphocyte proliferation in the spleen and inguinal lymph nodes of MOG-immunized mice. Suppression of peripheral immune cell CD4+ and CD8+ T cell proliferation at 5 and 12 days correlated with reductions in clinical behavior. EAE mice treated with OGF for 15 days displayed elevated Th1 and Th17 cells; no subpopulations of Th2-specific T cells were noted.

**Conclusions:**

OGF or LDN repress proliferation of CD4+ and CD8+T cells and B220+ B lymphocytes in the spleen and lymph nodes of immunized mice within a week of immunization. These data provide novel mechanistic pathways underlying the efficacy of OGF and LDN therapy for MS.

## Background

Peripheral T and B cell proliferation represents a hallmark of immune response to an antigen. In autoimmune disorders such as multiple sclerosis (MS), the self-antigen is unknown. The animal model of MS, experimental autoimmune encephalomyelitis (EAE), results from injection of small fragments of proteins associated with myelin such as myelin oligodendrocytic glycoprotein (MOG_35–55_) or polylipoprotein (PLP_139–155_)[1,2]. MOG_35–55_ immunization causes a chronic progressive form of EAE in C57Bl/6 mice, whereas PLP induces a relapse-remitting form of EAE in SJL mice [3-5]. Initial phases of development of both diseases are reliant on proliferation of T and B lymphocytes. The pathophysiology of both EAE and MS involves proliferation of CD4+ and CD8+ T lymphocytes, followed by CD4+ T cell-mediated demyelination [5,6]. Further proliferation of B cells and other T-cell subsets derived from secondary lymphoid tissues provide the basis for the inflammatory responses associated with EAE/MS [5-19].

Many of the FDA approved therapies for MS target CD4+ T cell signaling pathways, conversion of CD4+ T cells to T helper cells (Th1 and Th2), and transition of Th1- cells to Th17-T cells [4,10,11]. However, many of the disease-modifying therapies that target T cell proliferation are not completely successful, and new immune-cell mediated treatments have utilized B cell replication, as well as redistribution of T cell subpopulations, for their approach to stabilizing disease [8,10]. In response to immunization, antigen presenting cells such as dendritic cells secrete a variety of cytokines that stimulate CD4+ T cells to become differentiated into Th1, Th2, or Th17 through a process known as polarization [5]. Th1 cells are often considered pro-inflammatory, respond to IL-12, and produce IFN-γ, which enhances the pathway of CD4+ T cell proliferation and inflammation [5,15,16]. Th2 cells are considered to be anti-inflammatory because of the specific cytokines associated with them, including IL-4 shown to decrease disease severity. IFN-γ is known to inhibit Th2 polarization and thus exacerbate immune-responses to EAE [17]. Preproenkephalin mRNA has been detected, along with neuropeptides leucine enkephalin and methionine enkephalin (also termed OGF) in CD4+ Th1 and Th2 lymphocyte subpopulations [18]. However, the enkephalin prohormone was not required for Th1 differentiation. Transition of Th1 cells to the Th17 subpopulation, or increased Th17 subpopulations, are considered part of a “repair” mechanism and response of EAE pathogenesis [19].

The opioid growth factor (OGF) – OGF receptor (OGFr) pathway is a regulatory pathway involved in homeostatic cell replication. The OGF-OGFr pathway has been implicated to regulate immune-activated cell proliferation in a number of diseases including MS and animal models of EAE [20-26]. OGF, chemically termed [Met^5^]-enkephalin, is an inhibitory growth factor that binds to OGFr in a selective manner to upregulate cyclin-dependent inhibitory kinases and thus suppress replication. Low doses of naltrexone (LDN) work by biofeedback mechanisms to stimulate endogenous peptide and receptor release, thus providing enhanced inhibitory action. OGF and LDN therapies have been examined in both chronic progressive and relapse-remitting models of mouse EAE [20-26]. Systemic injections of OGF or LDN initiated at the time of disease induction by MOG_35–55_ demonstrated markedly less severe clinical signs of disease and reduced spinal cord neuropathology in C57Bl/6 J mice [20-22]. OGF treatment initiated at the time of established progressive disease reversed the course of EAE within 6 days [23]. Evaluation of spinal cord tissues demonstrated reduced numbers of activated astrocytes, microglia/macrophages, and damaged neurons, as well as decreased demyelination [23]. OGF and LDN therapy of established relapse-remitting EAE whereby PLP_139–151_ is injected into SJL/J mice and after 2 consecutive days of clinical signs of disease, mice are injected with OGF or LDN, revealed diminished peak disease scores for both treatments, and a significant number of mice experiencing partial, if not complete, remissions [24-26]. These data support the role of endogenous opioid suppression of EAE progression.

Previous work has shown that T and B lymphocytes isolated from normal mice, and stimulated in culture to divide, had significantly reduced proliferation following administration of OGF or LDN [27,28]. OGF inhibited lymphocyte cell replication in a dose-dependent, reversible and receptor mediated manner. Reduction of OGFr by siRNA technology prevented OGF’s inhibitory action. Moreover, no other exogenous and endogenous opioid peptides had an effect on inhibiting T or B cell replication in culture [27,28]. These *in vitro* data, along with the behavioral consequences of OGF or LDN on EAE mice, support the role of the OGF-OGFr axis as an important regulation pathway in early phases of EAE.

In adaptive immune diseases such as EAE, T cell expansion occurs in a dynamic manner which influences expression of clinical disease and response to immunotherapy [29-34]. The present experiments investigate how OGF or LDN alter early phases of immune response in EAE, and study whether endogenous opioids such as OGF selectively inhibit the proliferation of one or more subpopulations of T lymphocytes. These studies provide an improved understanding of mechanistic pathways involved in repression of MS by LDN or OGF therapy.

## Results

### Behavioral profile

**Figure 1:**
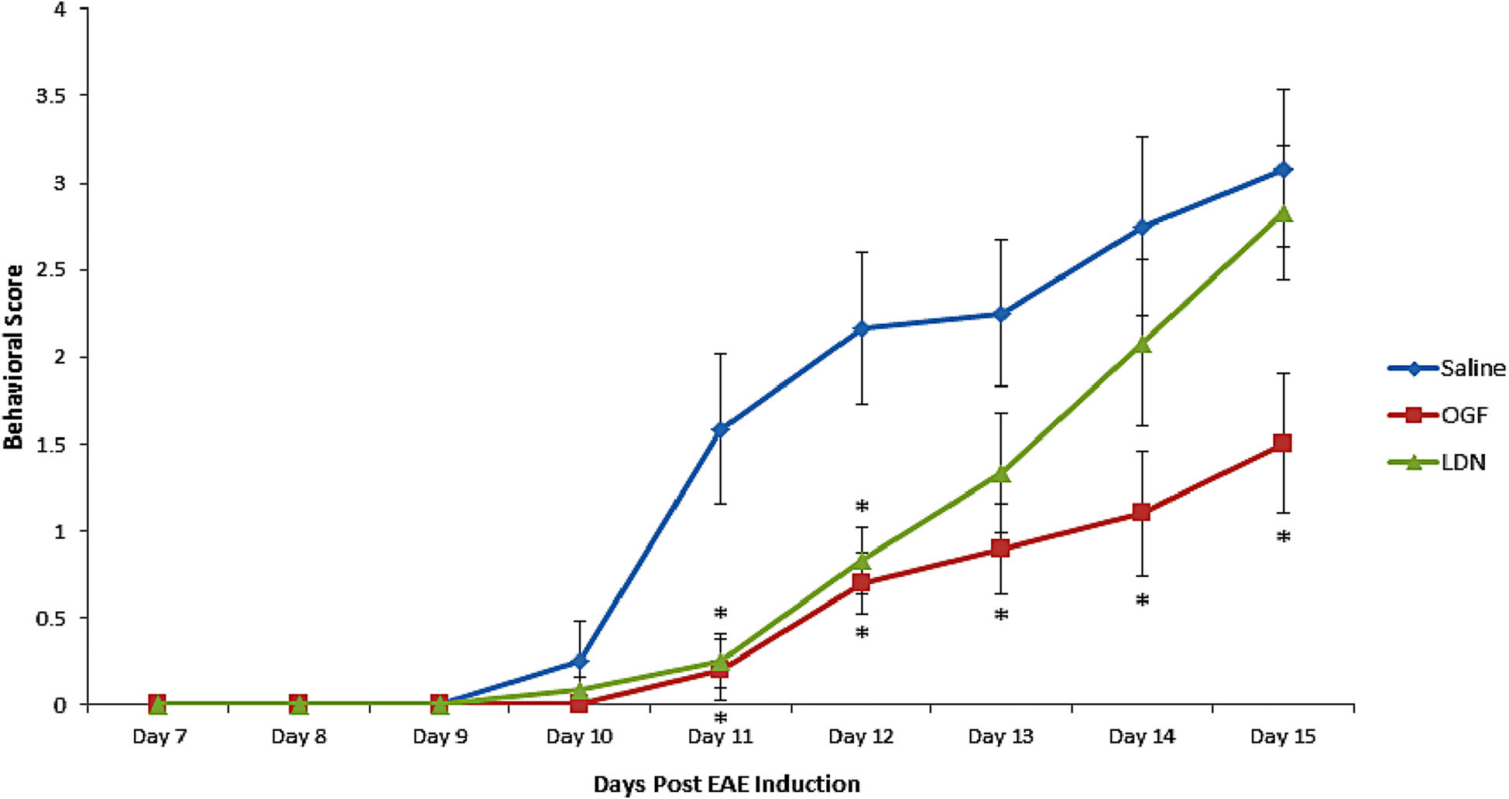
Behavior of mice immunized with myelin oligodendrocytic protein (MOG) to induce EAE and treated with OGF or LDN. Behavioral scores of mice immunized with MOG_33–55_ and treated daily with either 10 mg/kg OGF (OGF) or 0.1 mg/kg naltrexone (LDN); control mice received intraperitoneal injections of 0.1 ml saline (saline). Mice were evaluated daily and scored (0–10 scale) by multiple observers, with one masked to treatment. Values represent means ± SEM; N = 5 or 6 mice/group. Significantly different from saline-injected mice at p < 0.05 (*).

Mice immunized with MOG_35–55_ were observed daily and behavior scored by an observer masked to the identity of the mouse. Clinical disease appeared on day 9 or 10 for all mice (Figure 1). By day 11, mice receiving OGF or LDN beginning at the time of disease induction had significantly reduced behavioral scores, with an average of 0.5 or less for both groups in comparison to saline-treated mice with mean scores of 1.5 and 2 on days 11 and 12, respectively. By day 15, saline-treated EAE mice had behavioral scores of 3 indicating some paralysis, whereas mice in the OGF group had a mean disease score of 1.5. The reductions in overall disease scores were less that those reported for larger groups of mice [22].

### Total lymphocyte number in non-immunized, normal mice

The average numbers of lymphocytes isolated from the spleens of control, non-immunized C57Bl/6 mice on days 5, 12, and 15 were 4.9 x 10^7^, 5.8 x 10^7^ and 6.2 x 10^7^, respectively. On day 5, the average number of lymphocytes recorded in the inguinal lymph nodes of normal, non-challenged mice was 4.1 x 10^5^ (a 2.5 fold increase in MOG-stimulated mice). On days 12 and 15, the average number of lymphocytes in normal inguinal nodes ranged between 3.4 x 10^5^ and 4.1 x 10^5^.

### Total lymphocyte number in spleens of MOG-immunized mice

**Figure 2:**
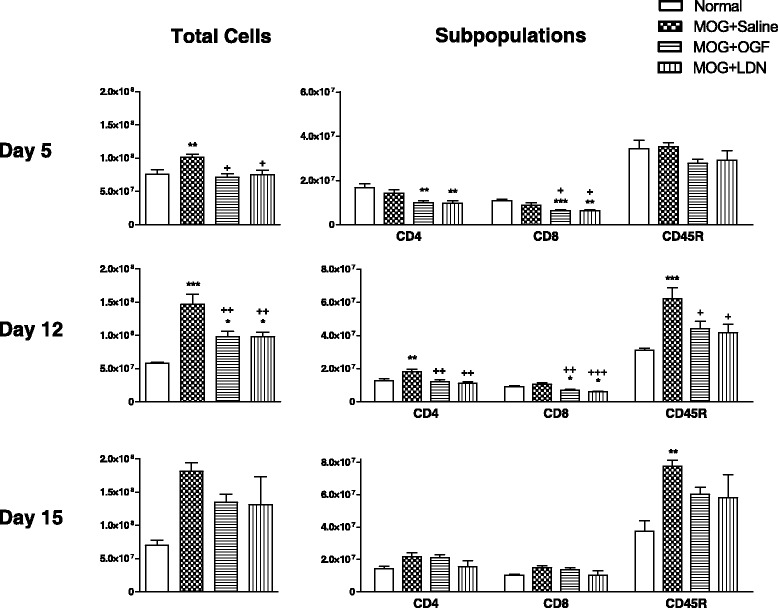
Effects of OGF or LDN on splenic lymphocytes from EAE mice. Lymphocytes isolated from spleens of C57BL/6 mice immunized with MOG_35–55_ and injected daily with 10 mg/kg OGF (OGF), 0.1 mg/kg naltrexone (LDN) or 0.1 ml saline (saline); normal mice received saline at each injection point. Splenocytes from 3–4 mice per treatment at 5 and 8 days were isolated, labeled, and sorted by flow cytometry. Values represent means ± SEM. Significantly different from normal mice at p < 0.05 (*) or p < 0.001 (***), and from saline-treated immunized mice at p < 0.05 (+) or p < 0.01 (++).

Splenic lymphocytes were isolated from non-immunized and MOG-immunized mice (EAE) on days 5, 12, and 15, and total cell number was recorded by a hemacytometer (Figure 2). Five days following immunization, normal C57BL/6 mice had approximately 76 million lymphocytes in the spleen in comparison to an average of 101 million lymphocytes in MOG-EAE mice (Figure 2). By day 12, MOG-immunized mice had enlarged spleens with 147 million lymphocytes in comparison to 58 million in non-immunized mice (p < 0.001). On day 15, lymphocyte number increased to 181 million in the immunized mice and 70 million in the non-challenged animals.

MOG-immunized mice received OGF or LDN beginning at the time of immunization and continued daily for 15 days (Figure 2). Within 5 days of treatment, both OGF- and LDN-treatment reduced the total number of lymphocytes in comparison to MOG-immunized mice; MOG + OGF mice had 71 million cells, and MOG + LDN animals had 75 million splenocytes, in comparison to approximately 101 million cells in MOG + Saline mice. On day 12, total lymphocyte number was decreased approximately 34% for both groups of MOG-immunized mice receiving either OGF or LDN; treated mice had approximately 97 million splenocytes in comparison to 147 million counted in MOG + Saline animals. On day 15, all groups of immunized mice had between 131 and 181 million total lymphocytes in the spleen.

### Subpopulations of splenic lymphocytes in OGF- and LDN-treated MOG-immunized mice

Flow cytometric analyses of CD4+ and CD8+ T cells, as well as B lymphocytes in the spleens of immunized mice revealed that OGF and LDN reduced immune-stimulated T cell proliferation at 5 and 12 days following MOG immunization (Figure 2). B cell number was inhibited by OGF and LDN on day 12.

Within 5 days of treatment with exogenous OGF or LDN, CD8+ T lymphocyte subpopulations in MOG + OGF and MOG + LDN mice were reduced by 29% to 32% from that recorded for MOG immunized saline-treated mice (Figure 2). CD4+ T cells in MOG + OGF or MOG + LDN groups were reduced by approximately 30% from that of MOG + Saline mice. B cell number was comparable for all groups of immunized mice and non-stimulated animals.

After 12 days of treatment, MOG-immunized mice receiving saline had approximately 18 million CD4+ splenic T lymphocytes, a 41% increase over non-immunized animals (Figure 2). Animals receiving OGF or LDN had 32-37% reductions in the number of CD4+ T cells relative to the number recorded for MOG + Saline mice. The subpopulation of CD8+ T cells in the spleens of MOG + OGF and MOG + LDN mice were reduced 35-42% relative to MOG + Saline mice, as well as reduced relative to normal mice. B cell number was elevated to 62 million, a 2-fold increase in comparison to the 30 million B-cells recorded in non-immunized normal mice. OGF and LDN treatment markedly reduced the number of CD45 stained B220+ B cells by approximately 29% from the saline-injected MOG mice.

On day 15 following immunization and treatment, OGF or LDN treatment had no effect on subpopulations of T cells isolated from the spleen; CD4+ and CD8+ T cell number ranged between 10 and 21 million for all treatment groups, including normal mice. B cell number in the spleen was elevated to 77 million in immunized mice from 38 million recorded for normal animals. OGF and LDN treated groups had approximately 60 million B cells in the spleen.

### Right inguinal lymph node lymphocyte proliferation

**Figure 3:**
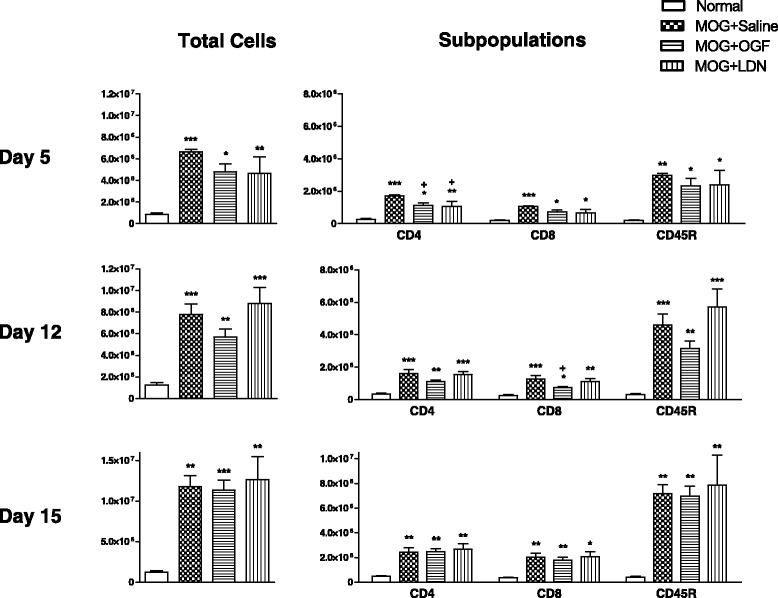
Inhibition of proliferation of T lymphocyte subpopulations in right inguinal nodes in EAE mice following OGF or LDN treatment. Total lymphocytes isolated from right inguinal nodes of mice immunized with MOG_35–55_ and injected daily with 10 mg/kg OGF (OGF), 0.1 mg/kg naltrexone (LDN), or 0.1 ml saline (saline); normal mice received saline at each injection point. Right nodes were activated following injections on day 0; lymphocytes from 3–4 mice per treatment at 5 and 12 days were isolated, labeled, and sorted by flow cytometry. Values represent means ± SEM. Significantly different from normal mice at p < 0.05 (*), p < 0.01 (**), and p < 0.001 (***), and from saline-treated immunized mice at p < 0.05 (+).

Mice were initially inoculated with MOG_35–55_ on the right side rendering the right inguinal node as a “draining node” on day 5. In comparison to 850,000 lymphocytes in a normal lymph node, stimulated mice had 6.6 million within 5 days of immunization (Figure 3). The total number of lymphocytes in the right, draining inguinal node of MOG immunized mice reached 11 million on day 15 in comparison to 1.2 million in normal mice. OGF-treated mice had total lymphocyte counts ranging from 4.8 to 11.4 Million, whereas LDN-treated MOG-immunized mice had total cell numbers in the right inguinal node of 4.6 to 12.6 million (Figure 3). OGF and LDN treatment did not reduce the overall cell number in the right inguinal node.

Analyses of T and B cell subpopulations using flow cytometry demonstrated that MOG-immunization increased CD4+ and CD8+ T cell number by 5- to 6-fold over normal mice, while B220+ cells detected by CD45 antibody were increased 15-fold in the right inguinal node within 5 days of injection of MOG (Figure 3). OGF and LDN treatment reduced CD4+ T cell number in the right inguinal node of MOG mice by 35 and 41%, respectively, on day 5. Although CD8+ and CD45+ specific T and B cells, respectively, were reduced by OGF and LDN, values were not significantly different.

After 12 days of immunization, and 7 days after direct injection near the right inguinal node, the right inguinal node of MOG + Saline mice had 4.5-fold more CD4+ T cells than recorded in nodes of non-stimulated animals. OGF or LDN treatment did not alter CD4+ subpopulations of lymph nodes cells. However, OGF reduced CD8+ T cells in the right lymph node by 43%, and LDN reduced cell number by 10%, in comparison to values reported for MOG + Saline mice. The subpopulation of B220+ B cells, as detected by CD45 antibodies, was increased 9- to 14- fold in the immunized groups relative to non-stimulated mice. Although OGF reduced B cell subpopulations in the right lymph node by 33%, values did not differ significantly from the MOG + Saline group; LDN treatment did not alter CD8+ T cell or B cell subpopulations.

Approximately 2 weeks after immunization (day 15), the total number of lymphocytes in the right inguinal node of immunized mice was approximately 5-fold that of non-stimulated animals, and comparable among all groups of MOG_35–55_ inoculated mice. Subpopulations of T cells were elevated approximately 5-fold and 6-fold respectively for CD4+ and CD8+ T cells, and more than 17-fold for CD45 positive B cells. OGF and LDN did not alter the subpopulation distribution in the right inguinal node at this time.

### Left inguinal lymph node lymphocyte proliferation

**Figure 4:**
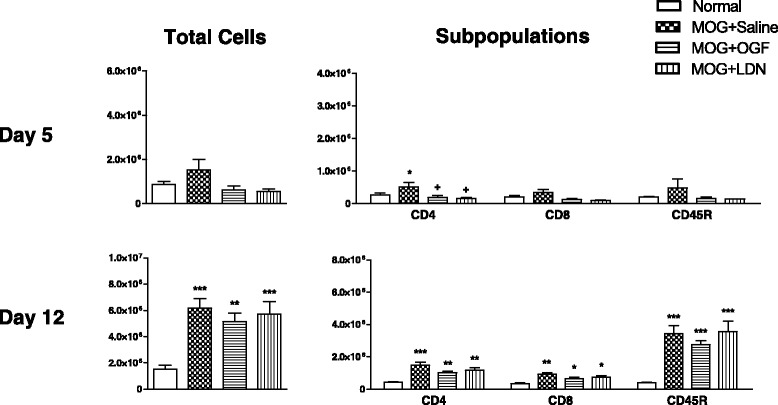
Inhibition of proliferation of T lymphocyte subpopulations in left inguinal nodes in EAE mice following OGF or LDN treatment. Total lymphocytes isolated from the left inguinal nodes of mice immunized with MOG_35–55_ and injected daily with 10 mg/kg OGF (OGF), 0.1 mg/kg naltrexone (LDN), or 0.1 ml saline (saline); normal mice received saline at each injection point. The left inguinal node was considered “draining” on day 12, 5 days following injection on the left flank. Lymphocytes from 3–4 mice per treatment at 5 and 12 days were isolated, labeled, and sorted by flow cytometry. Values represent means ± SEM. Significantly different from normal mice at p < 0.05 (*), p < 0.01 (**), and p < 0.001 (***), and from saline-treated immunized mice at p < 0.05 (+).

The second inoculation of MOG_35–55_ was on the left side on day 7 which stimulated the left inguinal node to become activated by day 12. Thus, on day 5, the total number of lymphocytes in the left inguinal node was comparable between all treatment groups, and ranged between 500,000 to 1.5 million cells (Figure 4). Subpopulations of T and B lymphocytes ranged from 100,000 to 500,000 cells. Nonetheless, OGF treatment inhibited cell replication of the CD4+ T cells by 64%; with only 185,000 cells recorded from MOG + OGF mice in comparison to 516,000 cells in the MOG + Saline animals. LDN reduced CD4+ T cells by 69% in comparison to MOG + Saline cell numbers. Although CD8+ T cells were reduced more than 50% by both OGF and LDN, values were not significantly different. CD45 stained B220+ B cell number ranged between 143,000 and 479,000 cells on day 5.

On day 12, cell number in the left inguinal node increased in the immunized mice to 5.1 – 6.1 million total lymphocytes (Figure 4). Although immunization significantly increased the number of CD4+ and CD8+ T cells, as well as CD45+ B cells, the number of lymphocytes in the left inguinal node was not inhibited by OGF or LDN.

By day 15, total left inguinal node cell number increased to greater than 10 million, in comparison to 1.2 million cells in the non-immunized mouse. Subpopulations of CD4+ T cells increased to 2 million in MOG-stimulated mice relative to less than 500,000 in the left inguinal node of normal mice. Similar profiles for expansion of CD8+ T cells were noted in the MOG-immunized mice; LDN and OGF had no effect on cell proliferation at 15 days.

### Intracellular distribution of CNS derived lymphocytes

**Figure 5:**
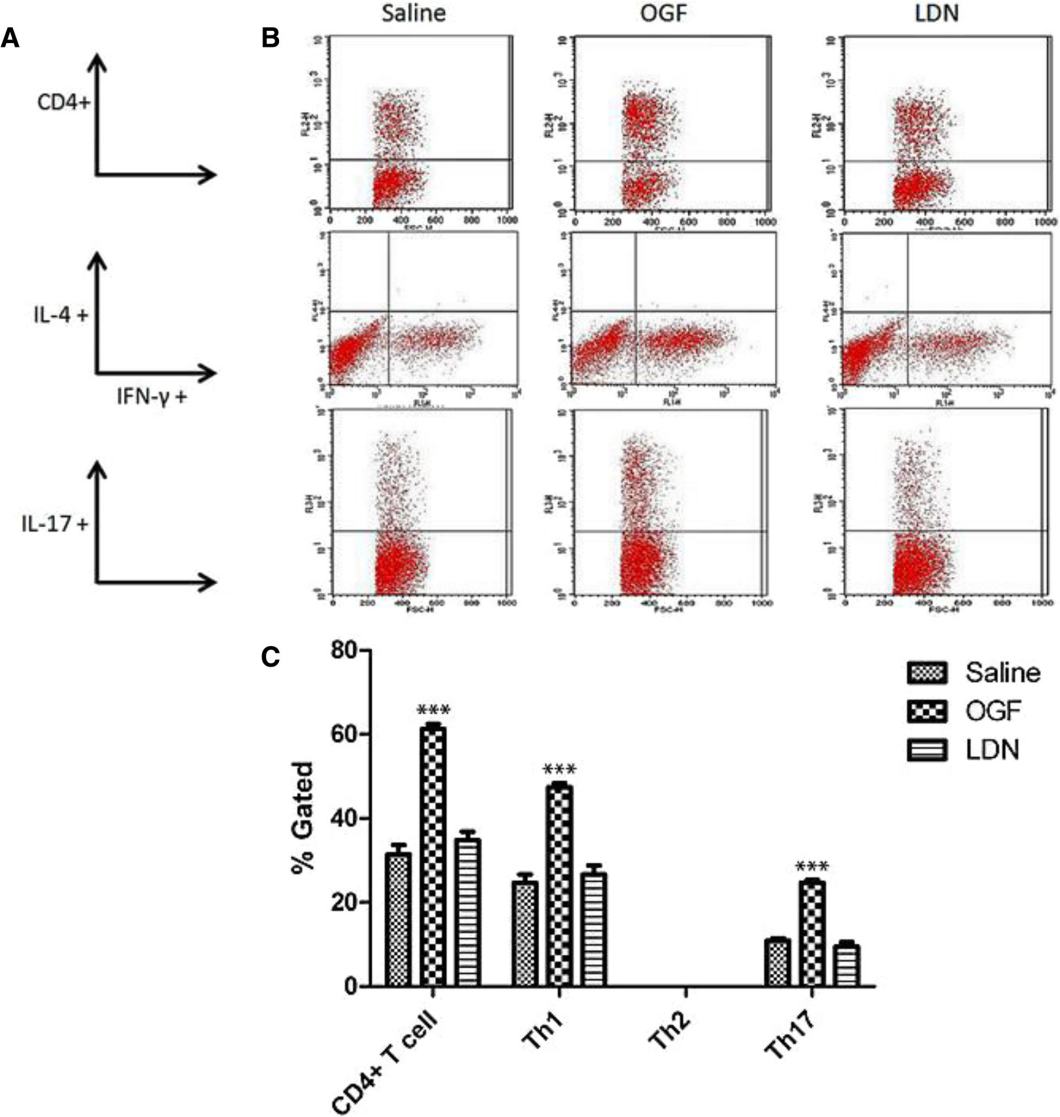
Differential T cell subpopulations in spinal cord of EAE mice treated with OGF or LDN. Differential cell counts of lymphocytes isolated from spinal cords isolated from mice immunized with MOG_35–55_ and treated from the time of induction with 10 mg/kg OGF (OGF), 0.1 mg/kg naltrexone (LDN), or 0.1 ml saline (saline); normal mice received saline at each injection point. Representative FACS profiles of T-cell populations obtained by fluorescence-activated cell sorting. Lymphocytes were isolated from spinal cord and stained with markers for CD4+, Th1, Th2, and Th17T cells. (A) Parameters for representative flow cytometry dot plots. (B) Representative flow cytometry dot plots for OGF, LDN, and saline treated mice 15 days after immunization with MOG_35–55_. (C) Histograms represent the percentage (± SEM) of electronically gated cells obtained from flow cytometry; samples were run in triplicate. Little or no Th2 cells were detected. Significantly different from saline-treated mice at p > 0.001 (***).

CNS derived lymphocytes isolated from the lumbar spinal cord of MOG-immunized mice treated for 15 days with OGF, LDN, or saline were assessed by intracellular cytokine staining. Cell homogenates were labeled with markers for CD4+ T cells, as well as for cytokines that were expressed on Th1, Th2, and Th17 subsets of T cells (Figure 5A). From six spinal cords per group, 5.25x10^6^, 4.35x10^6^, and 2.85x10^6^ lymphocytes were collected from the saline, OGF, and LDN treated groups respectively. Lymphocyte subpopulations were counted and revealed that the number of CD4+ cells for saline, OGF, and LDN groups were 1,645,175, 2,663,500, and 990,000 cells respectively. IFN- (FITC-stained) cells totaled 1,291,850, 2,063,000, and 759,000 cells respectively for saline, OGF, and LDN treated mice. Th2 (IL-4, APC stained) cells numbered 855 for LDN treated mice and negligible/none for saline and OGF treated mice. The largest number of Th17 cells were recorded in OGF mice (1,071,000) in comparison to 573,000 for saline-treated mice, and 270,300 for LDN treated animals. Of the lymphocytes analyzed by flow cytometry, OGF treatment resulted in a 1.9-fold increase in the percentage of total lymphocytes that were CD4+ T cells relative to the number recorded for saline-treated MOG-immunized mice (Figure 5B, C). OGF treatment resulted in an increase in the ratio of double- stained CD4+/IFN-γ (Th1) and CD4+/IL-17 (Th17) cells in comparison to the number of cells gated in the MOG + Saline group. OGF exposure increased the percentage of Th1 and Th17 cell subpopulations by 1.9-fold and 2.2-fold, respectively, as compared to the saline group (Figure 5C). LDN treatment did not alter the ratio of CD4+ T cells or the number of Th1, Th2, and Th17 subsets. Th2 cells, often considered as “anti-inflammatory”, were not detected.

## Discussion

Enkephalins have always been considered to have immunomodulatory properties [30]. The role of T and B cell proliferation in mice with experimental autoimmune encephalomyelitis is of great interest, and many papers have shown a biphasic proliferative role in cells, with T cell proliferation increased early in the process of immunization, and then reduced several weeks following antigen stimulation [31-34].

The present study is the first to demonstrate the inhibitory effect of the endogenous opioid OGF on cell proliferation, and particularly on inhibition of T cell proliferation *in vivo*. Previous work in our laboratory revealed that *in vitro* stimulation of T cells by phytohemagglutinin and incubation with OGF diminished cell replication. Splenic cells isolated from normal mice and treated with 10 μg phytohemagglutin per ml of media were stimulated to proliferate; treatment with OGF reduced cell proliferation in a dose-dependent manner. The repressive effects of OGF on proliferation were mediated through OGFr, as reduction of OGFr protein by siRNA technology removed the inhibitory effect of OGF [35]. The present experiments expand on our *in vitro* data [27,28] that demonstrated the presence of both OGF and OGFr in lymphocytes, and demonstrate that *in vivo* manipulation of the OGF-OGFr axis alters proliferation of different subpopulations of T and B lymphocytes in peripheral lymph tissue and spinal cord. Our tissue culture studies on T and B cell proliferation reveal that the mechanism of OGF’s inhibitory activity on cell number is by reducing DNA synthesis, and not by enhancing apoptosis or necrosis [27,28]. Incorporation of tritiated thymidine into the splenocytes in cultures also demonstrated the inhibitory effect of OGF. Treatment of stimulated T and B cells with other opioid agonists such as leucine enkephalin, endomorphin 1, β-endorphin, dynorphin 1–13, or morphine had no effect on immune cell-specific proliferation. In fact, β-endorphin may enhance cell proliferation of immune cells through non-opioid receptor mechanisms [31].

Reports on whether opioid peptides alter T and B cell proliferation are often conflicting [36-38]. Differences in the studies as to whether opioids inhibit or enhance immune-cell replication *in vitro* depend of a number of experimental factors including variations in dosage, class of peptide (natural or synthetic), whether cells are stimulated or not, and length of treatment. OGF did not affect proliferation in unstimulated cells in culture, or splenic cells *in vivo*.

Most studies on T and B cell proliferation in mice with an autoimmune disease are focused on conversion of T_reg_ to T_eff_ cell types, or changes between Th1 and Th2 or Th17 [18,19]. In the present studies, mice challenged with MOG_35–55_ peptide displayed clinical signs of experimental autoimmune encephalomyelitis by day 11 or 12. Clinical signs of behavioral deficits were evident on days 11 and 12 in the LDN group and on days 11–15 for the OGF-treated group. The behavioral scores in this experiment were limited to a small number of mice in comparison to other studies where mean disease scores were 50 to 75% reduced in animals treated with OGF or LDN relative to saline-treated EAE mice [22]. The minimal effects of LDN on behavior most probably extend to T cell proliferation as well, suggesting that a more robust effect is possible and that animal variation is always evident in behavioral studies.

Day 15 appeared to be the peak day of proliferation of splenic lymphocytes, whereas cell replication in inguinal lymph nodes peaked on day 15. OGF and LDN inhibited CD4+ and CD8+ T cell subpopulations, as well as CD45 stained B cells relative to values from saline-treated MOG-immunized mice on day 12. The greatest effects of OGF and LDN treatment on inhibiting the proliferation of T and B cell subpopulations in lymph nodes occurred on day 5. Total lymphocyte numbers were inhibited by as much as 40% following only 5 injections of OGF in comparison to total immune cell numbers in MOG immunized mice. Direct injections of OGF, rather than endogenous OGF stimulated by LDN, were more effective at repressing B and T lymphocyte replication. LDN had minimal effect inhibiting B cells in this model of EAE. With regards to CNS derived lymphocytes, OGF stimulated Th1 cell proliferation suggesting that cell-mediate immunity and interferon-γ and interleukin (IL)-2 are upregulated. The lack of Th2 cells being detected in the spinal cord may reflect the course of disease and the early period of sample collection (15 days) as Th2 cells tend to proliferate to inhibit phagocytic-dependent inflammation. The presence of Th17, but not Th2 cells, does not necessarily indicate that there are no anti-inflammatory cells responding to the antigen presentation [39,40]. Th17 effector cells, along with Th1 cells, are polarized to cause inflammation. Mice lacking either Th1 or Th17 were not immunoprotected suggesting that neither subset alone is responsible for disease progression. The increased proliferation of Th1 cells might simply reflect the early stage of disease and the peak flair; the lack of effect by OGF might indicate that treatment with endogenous opioids shifts the timetable of disease earlier so that Th1 cells are degrading intracellular pathogens earlier in the process, moving forward with anti-inflammatory response. Additional studies would be required for a complete investigation of Th1 to Th17 transition.

## Conclusion

The OGF-OGFr pathway regulates proliferation of peripheral immune cells following stimulation by MOG_35–55_ antigen. Exogenous OGF or endogenous OGF following LDN treatment inhibited CD4+ and CD8+ T and B cell replication within the spleen and draining inguinal nodes within the first week of immune-related response, and in spinal cord tissue on day 15 following antigen stimulation. These data support that modulation of the OGF-OGFr pathway is an effective therapeutic paradigm for MS.

## Methods

### Animals and induction of EAE

The mouse model of chronic EAE was established in 6-8-week old, female C57BL/6 J (stock 000664) mice, purchased from Jackson Laboratories (Bar Harbor, ME). Mice were housed, 5 per cage, in rooms maintained at 21 ± 0.5°C with a relative humidity of 50 ± 10%, with a complete exchange of air 15–18 times per hour and a 12 hour light–dark cycle with no twilight. Food and water were available *ad libitum*. All experiments were conducted in accordance with National Institute of Health guidelines on animal care and were approved by the Penn State Hershey Institutional Animal Care and Use Committee.

To induce chronic progressive EAE, each mouse received 400 μg myelin oligodendrocyte glycoprotein (MOG_35–55_) dissolved in 0.1 mL sterile phosphate buffered saline (PBS); 750 μg mycobacterium tuberculosis (H37RA, Difco Laboratories, Detroit, MI) was added to 0.15 mL incomplete Freund’s adjuvant (Sigma-Aldrich, St. Louis, MO) to create complete Freund’s adjuvant (CFA). Equal volumes of the PBS containing MOG_35–55_ and CFA were emulsified by vortexing. Mice were lightly anesthetized and injected subcutaneously on the right flank with 0.2 mL of the MOG_35–55_ emulsion (Peptides International, Louisville, KY). A second injection of MOG emulsion was injected in the left flank one week later. In addition, animals received an intraperitoneal injection of 500 ng pertussis toxin (List Biological Laboratories, Campbell, CA) dissolved in 0.2 mL PBS on days 0 and 2 of the study. Normal control mice were anesthetized and received sterile PBS injections of equal volume in place of MOG and pertussis immunizations. Mice were lightly anesthetized with 3% isoflurane (Vedco, Inc., St. Joseph, MO) for antigen and *M. tuberculosis* injections.

At the time of disease induction some mice were randomized to receive daily intraperitoneal injections (0.1 mL) of either 10 mg/kg OGF (Polypeptide Laboratories, Torrance, CA), 0.1 mg/kg naltrexone (LDN; SigmaAldrich, Indianapolis, IN), or an equal volume of sterile phosphate-buffered saline. All injections were given between 9.00 and 10.00 hr; anesthesia was not required for daily OGF or saline treatments. Animals were weighed weekly in order to adjust drug dosages.

### Behavioral observations

Animals were observed daily to evaluate the onset and progression of EAE [23]. Mice were placed individually on a clean, flat surface and behavior scored by 2 observers, one masked to treatment. Disease severity was evaluated on a 0 – 10 scale, with 0 representing no disease and 10 representing death due to EAE. Specifically, observations of tail tonicity, gait, righting reflex and limb function were scored and summed to provide a daily disease score [23]. Righting reflex tests were performed by placing the animal on its back and scoring the ability to return quickly to all four limbs. Limb function was scored by placing the mouse in an inverted position on a grid and observations of normal, weak, near paralysis or paralyzed limbs were assessed for each forelimb and hindlimb [23]. Mice with both hindlimb and forelimb paralysis received a score of 9, and death due to EAE was scored as 10.

### Lymphocyte isolation

For studies on chronic EAE, mice (3–5 per treatment group) were euthanized by cervical dislocation on days 5, 12, and 15 following immunization and lymphocytes processed according to published procedures [41-43]. Spleens and lymph nodes (right and left were kept separate) were removed, dissociated using 60-mesh stainless steel screens, and splenocytes or lymphocytes collected from their respective tissues. Red blood cells were lysed and cellular debris was removed by centrifugation. Lymph nodes of normal, unimmunized mice were pooled. Isolated lymphocytes were resuspended in Iscove’s modified Dulbecco’s media supplemented with 0.075% fetal calf serum, sodium bicarbonate, and β-mercaptoethanol, and counted using a hemacytometer and trypan blue exclusion staining.

### Lymphocyte subpopulation proliferation

Lymphocytes were stained with anti-CD4-PE conjugated antibody (clone GK1.5), anti-CD8a APC-conjugated antibody (clone 53–6.7), and anti-CD45R-FITC conjugated antibody (Clone RA3-6B2) to detect CD4+, CD8+, and B220 lymphocytes respectively; all reagents were purchased from eBioscience [41-43]. Cells isolated from spleen or right and left lymph nodes were stained, resuspended in FACS buffer, and analyzed by flow cytometry on a Becton Dickinson FACScan flow cytometer. Cell suspensions were processed in triplicate. Dot plots were analyzed with FlowJo© software (TreeStar, Inc, Ashland, OR).

### Intracellular cytokine staining of CNS lymphocytes

On day 15, spinal cord tissue was extracted from euthanized mice and pooled for each treatment group (N = 6). Tissue was dissociated and lymphocytes collected at the interface of a 63% - 27% Percoll gradient, plated in Iscove’s Modified Dulbecco’s Medium (IMDM) and stimulated overnight with phorbol 12-myristate 13-acetate (20 nM and ionomycin (1 μM) in IMDM at 37°C. Brefeldin-A was added at a final concentration of 9 μg/ml and incubated for 6 hours. Fc receptors were blocked with 2.4 g2 Fc blocking reagent in 10% normal mouse serum, followed by extracellular staining with anti-CD4+ antibody conjugated to PE. Cells were then washed, fixed, and permeabilized (Fix & Perm kit, Invitrogen, Grand Island, NY) and stained with antibodies to Th1, Th2, and Th17 using FITC conjugated anti-IFN- γ, APC conjugated anti-IL-2, and PerCP Cy5.5 conjugated anti-IL-17, respectively (eBioscience, San Diego, CA) following modifications of published and manufacturer’s instructions [5,6,43]. Replicate samples of stained cells were processed in the Core Faculty using a FACSCalibur flow cytometer (BD Bioscience).

### Statistical analyses

Tissues from 2 or 3 mice per treatment group at each time were collected; flow cytometry runs were made independently for 2 separate experiments. Experiments were performed in duplicate or triplicate; 50,000 events were collected per run. All data and plots from the flow cytometer were analyzed using CellQuest Pre software. Numerical data were analyzed by GraphPad Prism (GraphPad Prism Software, La Jolla, CA) using one-way analysis of variance with subsequent comparisons made using Newman-Keuls tests.

## Abbreviations

CD4+: Cluster of differentiation 4
CD8+: Cluster of differentiation 8
LDN: Low dosages of naltrexone
MOG: Myelin oligodendrocytic glycoprotein
OGF: Opioid growth factor
OGFr: Opioid growth factor receptor
PLP: Proteolipid protein
Th: T helper cells
T_reg_: T regulatory cells
T_eff_: T effector cells
